# Analysis of Social Media Perceptions During the COVID-19 Pandemic in the United Kingdom: Social Listening Study (2019-2022)

**DOI:** 10.2196/63997

**Published:** 2025-07-30

**Authors:** Marzieh Araghi, Arron Sahota, Maciej Czachorowski, Kevin Naicker, Natalie Bohm, Katie Phillipps, James Gaddum, Erica Jane Cook

**Affiliations:** 1 Pfizer Ltd, Walton Oaks, Dorking Rd, Surrey, KT20 7NS, United Kingdom, 44 7305408118; 2 Ipsos, 3 Thomas More Square, London, United Kingdom; 3 School of Psychology, University of Bedfordshire, Luton, United Kingdom

**Keywords:** COVID-19, social media, perceptions, sentiments, United Kingdom, patient experiences, diagnosis, treatment, SARS-COV-2, coronavirus, respiratory, infectious, pulmonary, pandemic, Synthesio, data, Google, trends, health information, public health, search query, Ipsos, named-entity recognition

## Abstract

**Background:**

Social media listening can be leveraged to obtain authentic perceptions about events, their impact, guidelines, and policies. There has been to date no research that has examined the experiences of patients with COVID-19 from diagnosis to treatment using social media listening in the United Kingdom.

**Objective:**

This study aimed to assess public perceptions, insights, and sentiments throughout the patient journey from diagnosis to treatment during the COVID-19 pandemic.

**Methods:**

A comprehensive search query was designed to retrieve social media data that referred to COVID-19 and treatment. The search was conducted using the social media monitoring tool, Synthesio (Ipsos). Data were retrospectively collected for the period covering September 2019 to September 2022 from Twitter (subsequently rebranded X), Facebook, Instagram, and YouTube as well as 126 public forums (including Health Unlocked, Mums Net, The Student Room, and Patient Forums UK). Available data in the United Kingdom expressed in the English language were collected and filtered, generating a final dataset consisting of 31,319 posts from an overall initial dataset of 706,634 posts. Complimentary Google trend analyses of search terms mentioning COVID-19 treatments were also performed.

**Results:**

Social media posts related to COVID-19 symptoms accounted for 6% of overall posts, compared to 35% of posts related to testing, 25% of posts related to diagnosis, and 32% of posts related to treatment. Overall, the trend observed from social media posts relating to COVID-19 treatment extracted in Synthesio was largely congruent with the trend of COVID-19 searches on Google, indicating a potential relationship between public discourse and social media and internet search behavior.

**Conclusions:**

The findings from this study have the potential to inform decision-making regarding public health interventions, communication strategies, and health care policies in the United Kingdom during future public health emergencies.

## Introduction

The World Health Organization declared COVID-19 a global pandemic on March 11, 2020, with more than 118,000 confirmed cases in 114 countries and 4291 deaths [1]. In the United Kingdom, the first confirmed case of COVID-19 was reported on January 29, 2020 [2] with the first death recorded on March 5, 2020 [3]. To mitigate the spread of COVID-19, a national lockdown was implemented in the United Kingdom on March 23, 2020 [4,5].

Temporary closure of health care facilities because of the national lockdown introduced new challenges for patients with COVID-19 such as accessing health services and public health information. Dissemination of public health information and guidelines relating to preventive measures and vaccination by the UK government was challenged by the complexity of pandemic risk communication and the need to reach across the diverse UK population [6,7]. In such a scenario, social media platforms became a useful and vital substitute for sharing public health information, evidenced by the rapid increase in the number of internet users during this period in the United Kingdom. The social media platforms including Facebook, Instagram, Twitter (subsequently rebranded X), WhatsApp as well as videoconferencing surged in popularity [8]. At the peak of the pandemic in 2020, an estimated 79% of “eligible users” (those aged 13 years and older) were active on social media, and the number of users reached 3.8 billion worldwide [9]. Thus, social media has played a positive and indispensable role in providing health information to the public [10].

Although the importance of social media for disseminating vital public health information cannot be overestimated, a concurrent rise in health misinformation was also observed. Social media posts relating to the origin of the virus, its pathogenesis, and transmissibility saturated social media platforms worldwide in a phenomenon termed as “infodemic” [11]. Processing social media activities may offer insights for monitoring public experience about different stages of the COVID-19 pandemic and how the health guidelines are perceived despite the flood of digital misinformation.

In the United Kingdom, previous social media listening studies have explored topics including COVID-19 vaccine hesitancy [12-15], the experience with health care [16], and the impact of COVID-19 on mental health [17]. However, to date, there has been no research examining the experiences of patients with COVID-19 from diagnosis to treatment. This research aims to address this gap, to provide useful information on how social media can be most effectively harnessed by health authorities to disseminate important public health messages, particularly to the UK’s diverse population. The study described here was undertaken to collect and collate social media posts during the COVID-19 pandemic in the United Kingdom with the objective to assess public perceptions, insights, and sentiments throughout the patient pathway.

## Methods

### Search Strategy and Data Collection

To investigate discussions surrounding COVID-19 treatment on social media, we collected data from various social media platforms using a comprehensive search query (Table S1 in Multimedia Appendix 1). Data collection spanned from September 2019 to September 2022 and focused on capturing data from the United Kingdom, specifically English language content. Below, we outline our data sources and search strategy.

- Social media platforms and web-based forums: we used the proprietary social media monitoring tool, Synthesio [18] (Ipsos), to extract data meeting our search criteria from Twitter/X, Facebook, Instagram, YouTube, and public forums. Synthesio collects data from over 800 million data sources, but this search was restricted to just those located in the United Kingdom or social media users from the United Kingdom, resulting in inclusion of data from 126 public forums (such as Health Unlocked, Mums Net, The Student Room, and Patient Forums UK).
- Google Trends analysis: To complement the social media and forum data, we conducted Google Trends analysis, which allowed us to observe trends in public interest regarding COVID-19 treatments over time, again using search terms mentioning COVID-19 treatment.

This approach yielded a dataset of 709,634 posts.

### Data Cleaning and Processing

Following the search, the generated dataset of 709,634 posts covering September 2019 to September 2021 was refined via a multistep process.

The steps are listed in Textbox 1.

Textbox 1. **Summary of data cleaning and processing procedures.**

1. Irrelevant content removal: the first step involved filtering out irrelevant posts to minimize noise and bias in the dataset, reducing the dataset to 325,770 posts. This included:
  - Automated filtering: We used Synthesio’s built-in “noise filters” to automatically remove posts categorized as job offers, vulgar content, scams, fraud, financial news, advertisements, content and games, and autoposting.
  - Manual removal: We further refined the dataset by manually removing posts identified as spam, advertisements, and news blog articles during a review of the dataset.
2. Duplicate removal: to avoid overrepresentation of specific topics or sentiments, we identified and removed duplicate posts or Retweets. Non-COVID-19 and treatment-related posts were also excluded, reducing the dataset to 107,796 posts.
3. Relevance labeling and machine learning: to enhance data quality, we implemented a machine learning–based approach:
  - Manual tagging: A random sample of 600 posts was manually classified as “relevant” or “irrelevant” to COVID-19 treatment discussions.
  - Model training: Using this manually tagged dataset, we trained a Convolutional Neural Network (CNN), a deep learning algorithm, to automatically categorize posts. The model was then applied to the remaining dataset. Model evaluation metrics (Table S2 in Multimedia Appendix 1) show an overall F1-score of 0.787. While the recall for relevant posts was low, potentially resulting in some relevant posts being filtered out, we prioritized accurately identifying and removing irrelevant content due to the dataset’s size.

### Text Processing and Normalization

To prepare the data for analysis, we performed several text processing steps using a Python pipeline [19]:

1. Sentence segmentation: dividing posts into individual sentences for a granular analysis.
2. Duplicate removal: identifying and removing duplicate sentences.
3. Tokenization: breaking sentences into words or noun phrases for analysis.
4. Lemmatization: converting words to their base forms to standardize text.
5. Additional cleaning: removing personally identifiable information, unifying text case, and eliminating stop words (eg, “at,” “on”).

### Selection of Themes and Conversations

For data categorization, we used Named Entity Recognition (NER) to classify unstructured text into predefined categories. We then identified key terms (~5000 in total) based on frequency (eg, “COVID” or “patient”). The list was manually reviewed and structured into a 3-tier hierarchy:

- Overarching group → Net → Codes and Terms
  - Example: Coronavirus → Variants → Delta, Omicron

Themes included symptoms, infection, testing, diagnosis, and treatment. Synonyms and related terms were grouped accordingly. Through this process, 89.7% of posts were categorized.

### Data Analysis

With all posts in the dataset categorized, we analyzed the data using 2 approaches listed in Textbox 2:

Textbox 2. **Summary of data analysis findings: overall trends and patient journey stages.**

- **Overall trends**
  - Frequency tables were generated to highlight dominant discussion themes.

- **Patient journey stages**
  - Posts were classified into symptoms, testing, diagnosis, and treatment categories using the Synthesio platform. Researchers manually reviewed and tagged posts to identify thematic trends [20].
  - Sentiment analysis [21] was conducted using a convolutional neural network–based model within Synthesio. Posts were classified as positive, negative, or neutral, with the model continuously refined via user feedback.

### Validity and Reliability

To cross-validate social media trends, we examined Google search data using AnswerThePublic [21], Google Trends [22], and Ahrefs [23]. These platforms provided insights into search volume and query types related to COVID-19 treatment, allowing us to compare social media discussions with public search behavior.

### Ethical Considerations

This study did not require human participant research review, as it did not meet the criteria for such oversight. All data were sourced from publicly accessible social media platforms. However, due to the inclusion of health-related content concerning COVID-19, additional privacy safeguards were implemented. Personally identifiable information was protected by using obfuscated identifiers; social media post IDs and usernames were redacted during data collection.

## Results

### Overview

**Figure 1.**
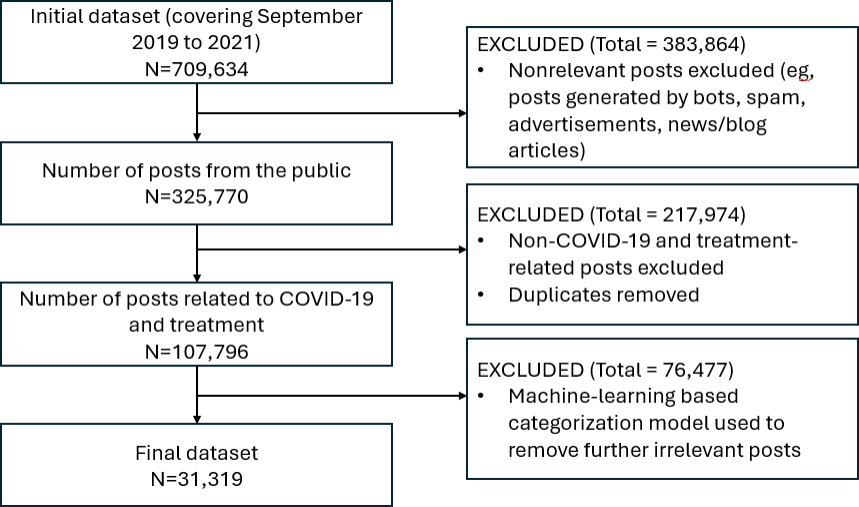
Flowchart of data process and analysis.

Following the search, the generated dataset of 709,634 posts covering September 2019 to September 2021 was refined via a multistep process (Figure 1). This comprehensive cleaning process resulted in a final dataset of 31,319 posts.

### Overall Trends on COVID-19 Social Media Activity

The final dataset is primarily composed of data from Twitter (subsequently rebranded X), with a minimal contribution from forums and other social media platforms such as Facebook, Instagram or YouTube.

**Figure 2.**
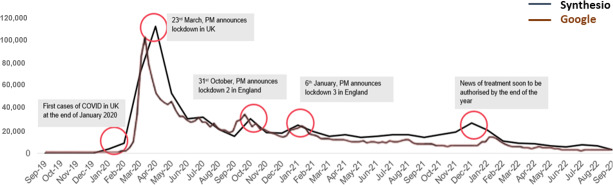
Total volume of posts around COVID-19 (Synthesio) and total volume of searches on COVID-19 (Google search). PM: Prime Minister.

Trends in social media discussions closely aligned with Google search trends. Social media posts emerged in December 2019, with the highest peak (107,796 posts) in March 2020, coinciding with the first UK lockdown [4]. Discussion volume decreased in late 2020 but spiked in response to COVID-19 waves, new variants, and treatment announcements (Figure 2). Synthesio data trends closely mirrored Google search patterns (Figure 2).

Notably, the Omicron variant was more prominent in social media discussions (3824 posts, 12.0%) compared to Delta (894 posts, 2.9%) (Figure S1 in Multimedia Appendix 1).

### The Patient Journeys

Thematic analysis was performed on the cleaned dataset (n=31,319) to further examine the search trends and volume on predetermined stages of the treatment pathway, that is, symptoms → testing → diagnosis → treatment.

#### Symptoms

Discussions about COVID-19 symptoms were prevalent, with 49% of posts mentioning general symptoms, with 17% specifically referring to “COVID-19 symptoms.” Frequently mentioned symptoms included cough, fever, fatigue, pain, anxiety, breathing difficulties, and respiratory issues [24].

#### Testing

**Figure 3.**
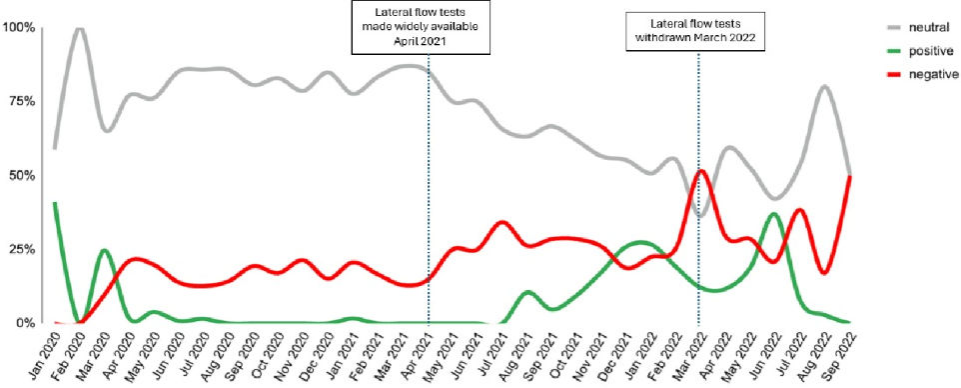
Sentiment trend of COVID-19 testing.

Conversations about COVID-19 testing accounted for 35% of discussions, with a primary focus on test accessibility, long wait times, and delays in receiving polymerase chain reaction (PCR) test results. Sentiment toward testing shifted over time. Initially, discussions were largely neutral, but sentiment became positive in July 2021 as PCR and rapid tests became widely available. However, a peak in negative sentiment occurred in March 2022, coinciding with the discontinuation of free rapid testing in the United Kingdom (Figure 3).

#### Diagnosis

**Figure 4.**
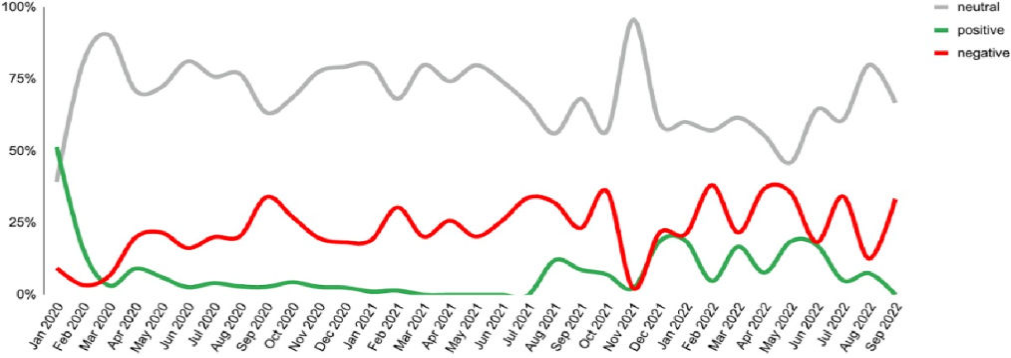
Sentiment trend of COVID-19 diagnosis.

The topic of COVID-19 diagnosis appeared in 25% of posts, with discussions primarily centered on test results and subsequent treatment options. Conversations about diagnosis peaked during major pandemic waves in October 2020, January 2021, and December 2021. Over time, sentiment became more positive, increasing from 2% to 19% by late 2021. However, negative sentiment remained prominent, ranging from 22% to 36% (Figure 4).

#### Treatment

Discussions about the treatment made up 32% of the dataset, often referring to medications in general terms such as “antiviral” (20% of total posts on treatment) or “medications” (39% of total posts on treatment). Misinformation was evident, as terms like “antimalarial” and “antibiotics” were frequently mentioned in the context of COVID-19 treatment (15% and 4% of total posts on treatment, respectively).

The analysis of top-searched terms and questions related to COVID-19 treatment on Google showed that eligibility for new COVID-19 treatment ranked highest, reflecting public interest in access to emerging therapies (Table S3 in Multimedia Appendix 1).

**Figure 5.**
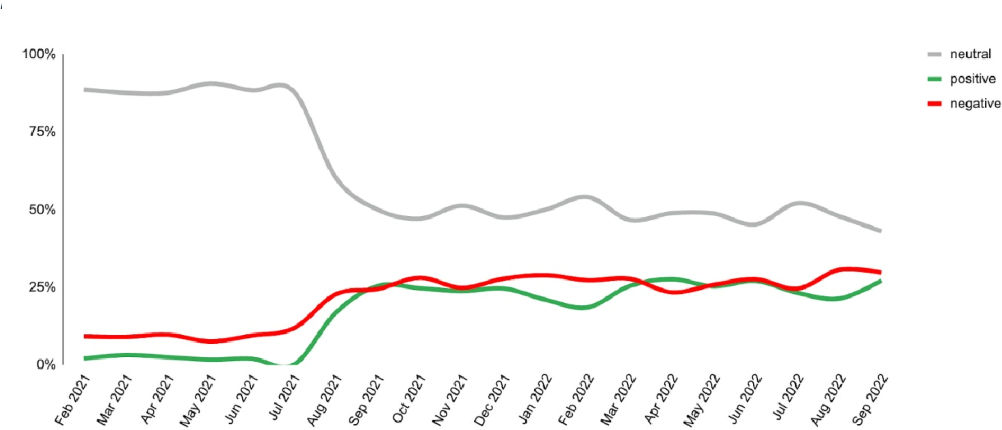
Sentiment trend of COVID-19 treatment.

The volume of treatment-related discussions peaked in December 2021, following announcements from the UK National Health Service regarding treatment availability. Sentiment analysis indicated that discussions were mostly negative (9%‐12%) until July 2021. From August 2021 onward, positive and negative sentiment balanced out, despite the availability of antiviral treatments from April 2022 [25] (Figure 5).

## Discussion

### Principal Findings

Our study examined public perceptions of COVID-19 symptoms, testing, diagnosis, and treatment through social media between September 2019 and September 2022. Public interest fluctuated, with peaks occurring during lockdowns, variant outbreaks, and treatment availability. The analysis of public perception of COVID-19 over time showed that the public attention fluctuated, with peaks occurring when lockdowns were introduced and when new variants emerged. The COVID-19 pandemic created an unparalleled sense of uncertainty and widespread health anxiety across the globe [26]. Despite or perhaps due to the vast amount of available information, uncertainty remains regarding the characteristics of the virus, as well as the emergence of new waves and variants. Studies show that information-seeking and knowledge-sharing practices increase rapidly in response to emergencies: since infectious diseases such as HIV or AIDS, acute respiratory syndrome, and Asian bird flu have emerged, the need for information by the public continues to increase [27-30]. Since social media is more frequently used than other media sources to acquire information on COVID-19, the government should have guidelines in place to ensure that reliable and accurate digital health information reaches diverse populations and those with lower levels of education.

Our findings show that COVID-19 symptoms represented 6% of social media expressions. People shared and sought validation for their symptoms, possibly reflecting anxiety, uncertainty, or a need for peer assurance. Posts related to symptoms ranged from less severe, like a cold, to potentially more severe, such as high fever, difficulty in breathing, and fatigue. Searches related to symptoms were closely linked to severity and age. In another study, a survey of over 2000 adults highlighted the awareness of main symptoms but not of others including skin rash, muscle and body aches, diarrhea, headache, nausea, and vomiting. Younger age groups in particular were least aware of COVID symptoms [31]. This lack of awareness may have led to increased anxiety and fear among the public, and to some extent a positive change in people’s health behaviors toward preventive measures [32].

Discussions about testing were common. The sentiment toward testing was neutral through the pandemic but turned positive from July 2021 and then became negative in the United Kingdom in March 2022. Diagnostic tests played a crucial role in COVID-19 management worldwide [33]. The public encountered challenges in accessing tests for diagnosis of COVID-19 infection particularly in the earlier days of the pandemic. Public frustration with testing delays was further aggravated as the pandemic progressed and test sites became overwhelmed. Major challenges encountered by health authorities included addressing the ever-increasing demand for testing and real-time PCR limitations (including being resource-intensive and time-consuming), which may have been the main source of public frustration with the test. Despite anxiety with regards to testing, social media activity eased during mid-2021, and public sentiment to COVID-19 testing also started turning positive. This aligned with the second phase in the evolution of the COVID-19 testing strategy which occurred several months into the pandemic, making diagnosis more affordable, easier, and faster than molecular tests [34].

The increase in negative sentiment in March 2022 could have been due to stopping free rapid COVID-19 tests in the United Kingdom. Ending free access to COVID-19 rapid tests could have contributed to health inequality, disproportionately affecting low-income and marginalized communities who may struggle to afford testing. This has the possibility of creating barriers to early detection and isolation, increasing the risk of transmission, particularly in high-risk settings [35]. Ensuring affordable, widespread testing is crucial for early intervention and outbreak control. Investing in community-based diagnostics, digital health solutions, and stronger public health data integration can improve disease monitoring and promote health equity, supporting better preparedness for future crises.

The diagnostic-related posts were less frequent and mostly involved sharing test results, which may reflect the normalization of COVID-19 infection over time. As COVID-19 became an ongoing part of daily life, public concern around diagnosis likely diminished, especially after the removal of widespread testing programs. With fewer policy changes and declining case severity, individuals who may have viewed COVID-19 as less urgent led to reduced engagement in discussions about diagnostic tools. This shift suggests that as disease becomes endemic, public interest in testing decreases, which has implications for maintaining awareness and ensuring continued access to diagnostic resources for effective disease monitoring.

Our analyses showed a large volume of queries about COVID-19 treatment, including its availability, effectiveness, and eligibility criteria. Initially, the public had a negative attitude toward possible side effects of antiviral treatments for COVID-19, and “rebound” was found to be the main concern for patients. Patients who tested positive for COVID-19 were also anxious regarding their eligibility to receive the available treatment. This concern was highlighted in the discussions due to the limited availability of the antiviral drugs, particularly for patients with mild-to-moderate symptoms. Difficulty accessing treatment and lack of access to information about treatment were also documented elsewhere as being particularly challenging for vulnerable communities in the United Kingdom throughout the pandemic, which may have influenced these sentiments [36].

### Comparison With Prior Work

Our findings align with previous studies showing that social media trends mirror Google search behavior [37]. Similar studies have confirmed that digital platforms effectively capture real-time public interest in health topics [38-41]. However, social media also contributed to misinformation, highlighting the importance of public health communication strategies [42]. Of note, the UK government and WHO had collaborative campaigns and created a series of social media infographics and messages to explain the safety profile of COVID-19 vaccines [43]. In addition, the British Broadcasting Company launched a successful project called “Stop the Spread” during May and June 2020; the purpose of which was to increase the public’s awareness of the magnitude of COVID-19 misinformation and motivate them to limit the spread and associated consequences.

### Limitations

One key limitation of this study is the demographic representation of social media users. Twitter data do not fully reflect the general population, and user characteristics, such as age, gender, and location could not be inferred. Although some geographic analysis was conducted, the findings did not show significant differences and were therefore not included in the study. Another limitation is the observational nature of the study. The comparison between social media trends and Google searches is descriptive rather than a statistical correlation analysis. To ensure clarity, we explicitly state this in the study to avoid any causal implications. Finally, there are constraints related to the data source. The use of Synthesio, a proprietary tool, limits the reproducibility of the study. In addition, while efforts were made to filter noise and spam, some irrelevant content may persist due to the inherent limitations of natural language processing techniques.

### Future Work

The nature of internet-based conversation about COVID-19 is rapidly changing, providing insights regarding the public’s thoughts and needs. Governments and public health authorities can leverage these insights use these insights to enhance crisis communication, combat misinformation, and promote vaccine uptake. By monitoring social media discussions, policy makers can identify emerging concerns and misconceptions, allowing them to tailor public health messaging accordingly. For example, during COVID-19 peaks, public uncertainty about symptoms, testing, and treatments could have been addressed through targeted social media campaigns to provide accurate information and alleviate confusion.

In addition, our findings highlight the role of social media in both spreading reliable information and amplifying misinformation. Health authorities should proactively track trending misinformation, implement fact-checking initiatives, and collaborate with platforms to promote credible sources. The success of initiatives like the WHO’s “Stop the Spread” campaign underscores the potential impact of coordinated efforts to counteract health misinformation and enhance public understanding.

Social media insights can also help inform vaccine communication strategies by identifying public concerns and hesitations regarding treatments and vaccinations. By analyzing discussions and trends, policy makers can develop targeted campaigns that address specific fears, correct misunderstandings, and promote informed decision-making. Integrating social media insights with traditional public health surveillance systems can further strengthen early detection of emerging health threats. By leveraging these real-time insights, governments can proactively engage with the public, reduce uncertainty, and build trust during health crises.

Future research could also build upon and deepen the types of analysis we conducted for this study. For example, a more comprehensive sentiment analysis could be conducted delving into a deeper range of emotions beyond the positive, neutral, and negative categorization used in this research [44,45], or further exploring how the different proportions of post related to each journey theme changed over time in line with previous studies [45,46]. We also note that while we did not analyze social media platform content individually against Google activity, future similar types of research could further investigate differences by social media platforms, Google activity, or both to clarify which platforms bear the most similarities in trends, particularly regarding conversations around health and health emergencies.

### Conclusions

Throughout our social listening analysis on several social media platforms, we had a promising overview of patients with COVID-19 journey from diagnosis to treatment. The study highlights the significant role of social media in shaping public perceptions of COVID-19 and underscores its potential as a tool for public health interventions. By leveraging social media analytics, policy makers can gain valuable real-time insights into public sentiment, allowing for more effective crisis communication, misinformation management, and targeted health campaigns. Future public health strategies should incorporate social listening tools to identify trends, address misinformation, and foster trust through clear and responsive communication.

## Supplementary material

10.2196/63997 Multimedia Appendix 1 Supplementary Materials.

## Abbreviations

CNN: convolutional neural network
NER: named entity recognition
PCR: polymerase chain reaction
