# Supplementary material for: Analysis of Social Media Perceptions During the COVID-19 Pandemic in the United Kingdom: Social Listening Study (2019-2022)
Source: JMIR Form Res. 2025 Jul 30;9:e63997. doi: 10.2196/63997 (PMC12310183; doi:10.2196/63997)
Supplement: Multimedia Appendix 1 [file formative-v9-e63997-s001.docx]

# Supplementary Materials

### Supplementary Table S1. List of search queries for COVID-19 discussion data collection

| (((((Corona OR Coronavirus OR Covid OR COVID19 OR CV19 OR “COVID?19” OR delta OR omicron) NEAR (treatment OR meds OR med OR medication OR treatable OR antiviral OR treat* OR prescr* OR pill OR tablet)) NOT “Chicago Med”)  OR  (Paxlovid OR (Ritonavir NEAR (Corona OR Coronavirus OR Covid OR COVID19 OR CV19 OR “COVID?19” OR delta OR omicron)) OR (Nirmatrelvir NEAR (Corona OR Coronavirus OR Covid OR COVID19 OR CV19 OR “COVID?19” OR delta OR omicron))))) |
| --- |

### Supplementary Table S2. Convolutional Neural Network Model Evaluation

|  | **Precision** | **Recall** | **F1-score** |
| --- | --- | --- | --- |
| 0 | 0.817 | 0.873 | 0.844 |
| 1 | 0.717 | 0.623 | 0.667 |
| Accuracy |  |  | 0.787 |

### Supplementary Table S3. Top searches and questions related to COVID-19 treatment

| **TOP QUESTIONS RELATED TO COVID TREATMENT** | |
| --- | --- |
| ***Question*** | ***Rank*** |
| Who is eligible for new covid treatment | 1 |
| What treatment is given to covid patients | 2 |
| What treatment is there for covid | 3 |
| How to get monoclonal antibody treatment for covid | 4 |
| What is the treatment for covid | 5 |
| Who is eligible for covid treatment | 6 |
| Long covid symptoms treatment what we | 7 |
| Long covid treatment what we know | 8 |
| What is the treatment for long covid | 8 |
| Who is eligible for community covid treatment | 8 |
| Long covid symptoms treatment what know | 9 |
| Long covid treatment what we so | 9 |
| Long covid symptoms treatment what far | 9 |
| What is the treatment of covid 19 | 9 |
|  | |
| *NOTE: Ahrefs, 17^th^ November 2021 – 17^th^ November 2022 “covid treatment”, UK only*  *An estimation of the average monthly number of searches for a keyword over the latest known 12 months of data* | |

### Supplementary Figure S1. Volume of discussions by variant

**
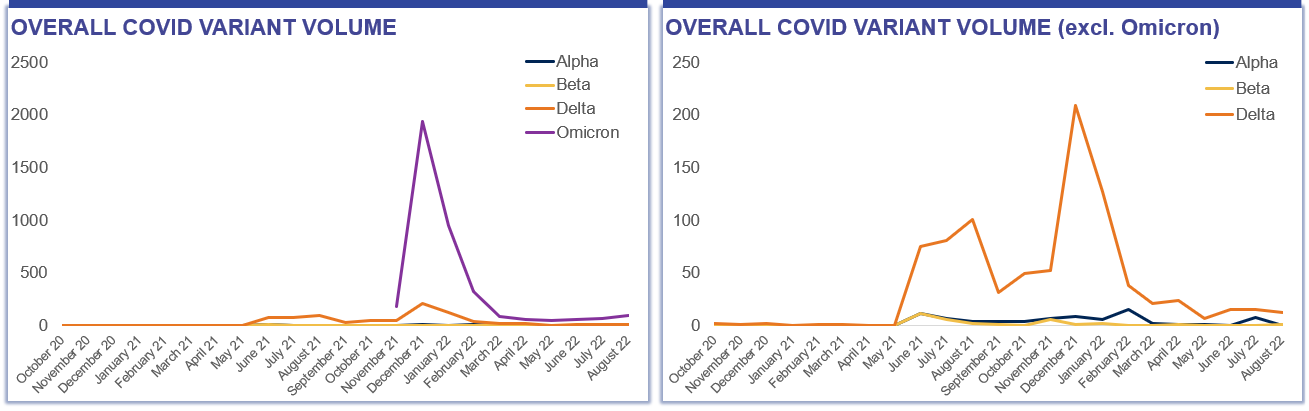
**
